# Molecular Characterization of Recombinant Human Inhibin α- and βB-Subunits and Activin A (βA-βA) Using Raman Spectroscopy

**DOI:** 10.3390/cimb48100980

**Published:** 2026-09-24

**Authors:** Weiyu Xiao, Yuzhen Dai, Sofia Martinez Gallardo Quijano, Domna G. Kotsifaki, Anastasia Tsigkou

**Affiliations:** 1 Gynecology & Oncology Research Lab, Division of Natural and Applied Sciences, No. 8 Duke Avenue, Kunshan 215316, China; weiyu.xiao@dukekunshan.edu.cn (W.X.);; 2 Photonics Lab, Division of Natural and Applied Sciences, Duke Kunshan University, No. 8 Duke Avenue, Kunshan 215316, China

**Keywords:** Raman spectroscopy, TGF-β superfamily, inhibins, INHA, INHBB (βB-subunit), activin A (βA-βA homodimer), label-free analysis, protein classification, disulfide bonds, endocrine signaling

## Abstract

Members of the transforming growth factor-β (TGF-β) superfamily, including inhibins and activins, are structurally related signaling proteins that regulate reproductive and endocrine physiology. Although these proteins share a conserved cystine-knot architecture, differences in subunit composition and molecular organization contribute to distinct biological activities and can complicate their analytical discrimination. Here, Raman spectroscopy was used to characterize recombinant human inhibin α-subunit (INHA), recombinant human inhibin βB-subunit (INHBB (βB-subunit)), and activin A (βA-βA homodimer) under identical experimental conditions using 532 nm excitation. Raman spectra were baseline-corrected, Savitzky–Golay-smoothed, and area-normalized. Distinct spectral features were observed across the 500–1800 cm^−1^ protein fingerprint region, particularly in the S–S stretching, aromatic amino acid residue, amide III, and amide I regions. The S–S stretching region showed the highest relative contribution in INHA, whereas differences in phenylalanine- and amide-associated bands further distinguished the three proteins. These findings show that Raman spectra may contain molecular information that can distinguish closely related inhibin/activin proteins under the investigated solid-state conditions. Raman spectroscopy therefore provides a label-free, complementary approach to the molecular fingerprinting of TGF-β superfamily proteins and may facilitate their molecular characterization.

## 1. Introduction

The transforming growth factor-β (TGF-β) superfamily comprises more than 30 structurally related signaling proteins that regulate diverse biological processes, including embryonic development, cell proliferation, differentiation, apoptosis, tissue remodeling, and endocrine homeostasis [1,2,3,4]. Within this superfamily, inhibins and activins are important regulators of reproductive physiology, particularly through their effects on follicle-stimulating hormone (FSH) secretion from the anterior pituitary. Inhibins are heterodimeric glycoproteins composed of a common α-subunit paired with either a βA- or βB-subunit, forming inhibin A (α-βA) and inhibin B (α-βB). However, activins are β-subunit dimers, including activin A (βA-βA), activin B (βB-βB), and activin AB (βA-βB), and generally promote FSH synthesis and secretion [3,4]. Together, these ligands contribute to regulation of the hypothalamic pituitary gonadal axis and follicular development [3,4].

Beyond reproductive physiology, dysregulated inhibin and activin signaling has been implicated in inflammation, fibrosis, tissue remodeling, and tumor progression in several tissues, including the ovary, prostate, breast, cardiovascular system, and endometrium [5,6,7]. Because biological activity is strongly influenced by subunit composition, receptor interactions, and ligand structure, distinguishing closely related inhibin and activin proteins is important for understanding their physiological and pathological functions.

Structurally, inhibins and activins share the conserved cystine-knot motif characteristic of the TGF-β superfamily, comprising a disulfide-stabilized core and extended β-strand regions that contribute to ligand stability and receptor recognition [8,9,10,11,12]. The presence of the inhibin α-subunit creates structural features that distinguish inhibins from activins and permits interaction with the co-receptor betaglycan, thereby contributing to antagonism of activin signaling [3,10,11,12,13]. Despite their conserved overall folds, differences in subunit composition, disulfide bond organization, dimer interface geometry, and conformational flexibility contribute to distinct biological activities [8,9,10].

The high structural similarity among TGF-β family proteins presents a challenge for analytical discrimination. In particular, related β-subunits share substantial sequence and structural similarity and adopt closely related cystine-knot folds. Conventional immunoassays, including ELISA and Western blotting, provide valuable information on protein abundance but depend on antibody specificity and may provide limited information about intrinsic molecular structure [3,12,13,14]. Mass spectrometry enables detailed molecular characterization but generally requires sample processing and may provide limited information about conformational states under the original measurement conditions [8]. Raman spectroscopy (RS) offers a complementary, label-free approach by probing intrinsic molecular vibrations related to protein composition and structure. Raman bands arising from disulfide bonds, aromatic amino acid residues, and peptide backbone vibrations in the amide I–III regions can provide information about cysteine environments, side-chain organization, and protein conformation [15,16,17,18]. These features are sensitive to the local chemical environment, hydrogen bonding, side-chain packing, and conformational constraints, making RS potentially useful for detecting subtle molecular differences among related proteins.

Although the cystine-knot scaffold is conserved across the TGF-β superfamily [19], individual ligands differ in disulfide topology, receptor binding interfaces, and conformational dynamics [19,20]. Such molecular differences may generate distinguishable vibrational signatures, particularly in spectral regions related to disulfide bonds, aromatic residues, and peptide backbone structures. To date, Raman-based comparative characterization of inhibin α- (INHA), inhibin βB-subunit (INHBB (βB-subunit)) and activin A (βA-βA homodimer) has received limited attention. Assessing whether their Raman spectral features enable reliable differentiation may provide a useful basis for their label-free identification and characterization.

In this study, we performed a Raman-spectroscopic analysis of recombinant INHA, INHBB (βB-subunit), and activin A (βA-βA homodimer). By combining Raman spectral analysis with heatmap visualization of normalized representative band intensities, we investigated whether intrinsic vibrational characteristics corresponding to disulfide bonds, aromatic amino acid residues, and protein backbone structures can distinguish closely related TGF-β superfamily proteins. This approach provides a complementary structural characterization method for use alongside established biochemical assays.

## 2. Materials and Methods

### 2.1. Protein Samples

Recombinant human inhibin α-subunit (INHA; Cat. No. ab280388) and recombinant Inhibin βB-subunit (INHBB (βB-subunit)); Cat. No. ab158764) were purchased from Abcam Ltd., (Cambridge, CB2 0AX, UK). Recombinant human activin A (βA-βA homodimer; Cat. No. 11344-AC) was purchased from R&D Systems (Minneapolis, MN, USA). All recombinant proteins were supplied at research-grade purity according to the manufacturers’ specifications. Lyophilized INHA and activin A were stored at −20 °C in sealed containers protected from moisture until analysis. INHBB (βB-subunit) was supplied in the manufacturer’s recommended storage buffer and stored at −80 °C until use. Samples were handled according to their supplied physical states and were not subjected to additional freeze–thaw cycles before Raman measurements.

### 2.2. Sample Preparation for Raman Measurements

Raman measurements were performed using the proteins in their commercially supplied form without additional chemical modification, labeling, or staining. Approximately 2 μg of each protein preparation was deposited onto a clean Raman-compatible glass microscope slide. Lyophilized INHA and activin A were analyzed directly after deposition, without reconstitution, to minimize additional sample-processing steps. INHBB (βB-subunit), which was supplied in the manufacturer’s storage buffer, was analyzed as-supplied. After deposition, samples were allowed to equilibrate under ambient laboratory conditions (22–25 °C) for 10 min before spectral acquisition. For each protein, Raman spectra were collected from five independent protein preparations, with measurements obtained from three spatially distinct locations on each deposited sample. The spectra from the three locations were averaged for each preparation, and the resulting spectra were averaged across the five independent preparations.

### 2.3. Raman Spectral Acquisition and Data Processing

Raman spectra were acquired using a confocal Raman microscope (Horiba Scientific, Kyoto, Japan) equipped with a motorized XYZ stage. Excitation was provided by a 532 nm continuous-wave diode laser focused onto the sample. Laser power at the sample plane was maintained below 15 mW to minimize local heating and photodegradation. Rayleigh scattering was suppressed using a notch filter, and Raman-scattered photons were dispersed using an 1800 line/mm grating and detected with a thermoelectrically cooled CMOS detector (Horiba Scientific, Kyoto, Japan). The spectrometer was calibrated before each measurement session using the silicon standard band at 520.7 cm^−1^. Spectra were collected over 300–2800 cm^−1^, with subsequent interpretation focused primarily on the protein fingerprint region. Each spectrum was acquired using a 15 s integration time with two accumulations. Background spectra from clean glass substrates acquired under identical experimental conditions were subtracted from the protein measurements.

Spectral preprocessing was performed using OriginPro 2025b (OriginLab Corporation, Northampton, MA, USA). Fluorescence background contributions were corrected using third-order polynomial baseline subtraction. The same baseline correction procedure was applied to all spectra. Raman spectra were then smoothed using a Savitzky–Golay filter with a fifth-order polynomial and a 50-point window to reduce high-frequency noise while preserving characteristic peak positions and relative intensity distributions. The same smoothing parameters were applied to all spectra. Total area normalization was subsequently performed over 300–2800 cm^−1^. Each spectrum was normalized by dividing the intensity at each Raman shift by the integrated area of the corresponding processed spectrum:

(1) $$I_{norm\left(f\right)}=\frac{I(f)}{\int_{300}^{2800}I\left(f\right)df}$$

where *I(f)* represents the baseline-corrected Raman intensity at Raman shift *f*. Total area normalization was used to reduce differences in overall signal intensity arising from variations in deposited sample amounts, laser focusing, and collection efficiency while preserving relative spectral features.

### 2.4. Heatmap Analysis

A heatmap of the normalized intensities of selected characteristic Raman bands, including the disulfide (S–S), phenylalanine (Phe), tyrosine (Tyr), and amide III regions, was generated in OriginPro 2025b to facilitate comparison of the spectral features among the proteins. The selected spectral regions contain characteristic Raman features corresponding to disulfide bonds, aromatic amino acid residues, and the protein backbone.

## 3. Results

### 3.1. Raman-Spectral Profiling of TGF-β Superfamily Proteins

Raman spectra of recombinant human INHA, INHBB (βB-subunit), and activin A were acquired under the same instrumental and acquisition conditions to compare their molecular vibrational signatures (Figure 1). Measurements were performed using the proteins in their commercially supplied forms, including the lyophilized preparations for INHA and activin A and the buffered preparation for INHBB (βB-subunit), which differed in physical state from the lyophilized preparations. The spectral preprocessing and analysis procedures were applied consistently across all protein preparations, as described in the Materials and Methods Section.

The three proteins exhibited characteristic Raman bands within the protein fingerprint region (500–1800 cm^−1^), including features corresponding to disulfide bonds, aromatic amino acid residues, and peptide backbone vibrations (Figure 1). The principal spectral regions examined were the S–S stretching region (500–550 cm^−1^), tyrosine-associated bands (~830–850 cm^−1^), the phenylalanine ring-breathing mode (~1000–1030 cm^−1^), the amide III region (1220–1300 cm^−1^), and the amide I region (1600–1700 cm^−1^). Assignments were based on previously reported Raman studies of proteins and biomolecules and are summarized in Table 1 [15,18,20,21,22,23,24,25,26,27,28,29,30,31]. Although INHA, INHBB (βB-subunit), and activin A share the conserved cystine-knot framework of the TGF-β superfamily, their Raman profiles differed in relative band intensity and spectral distribution. These observations may suggest that the Raman fingerprints of the three proteins contain distinguishable spectral features related to differences in molecular composition and structural organization.

**Figure 1:**
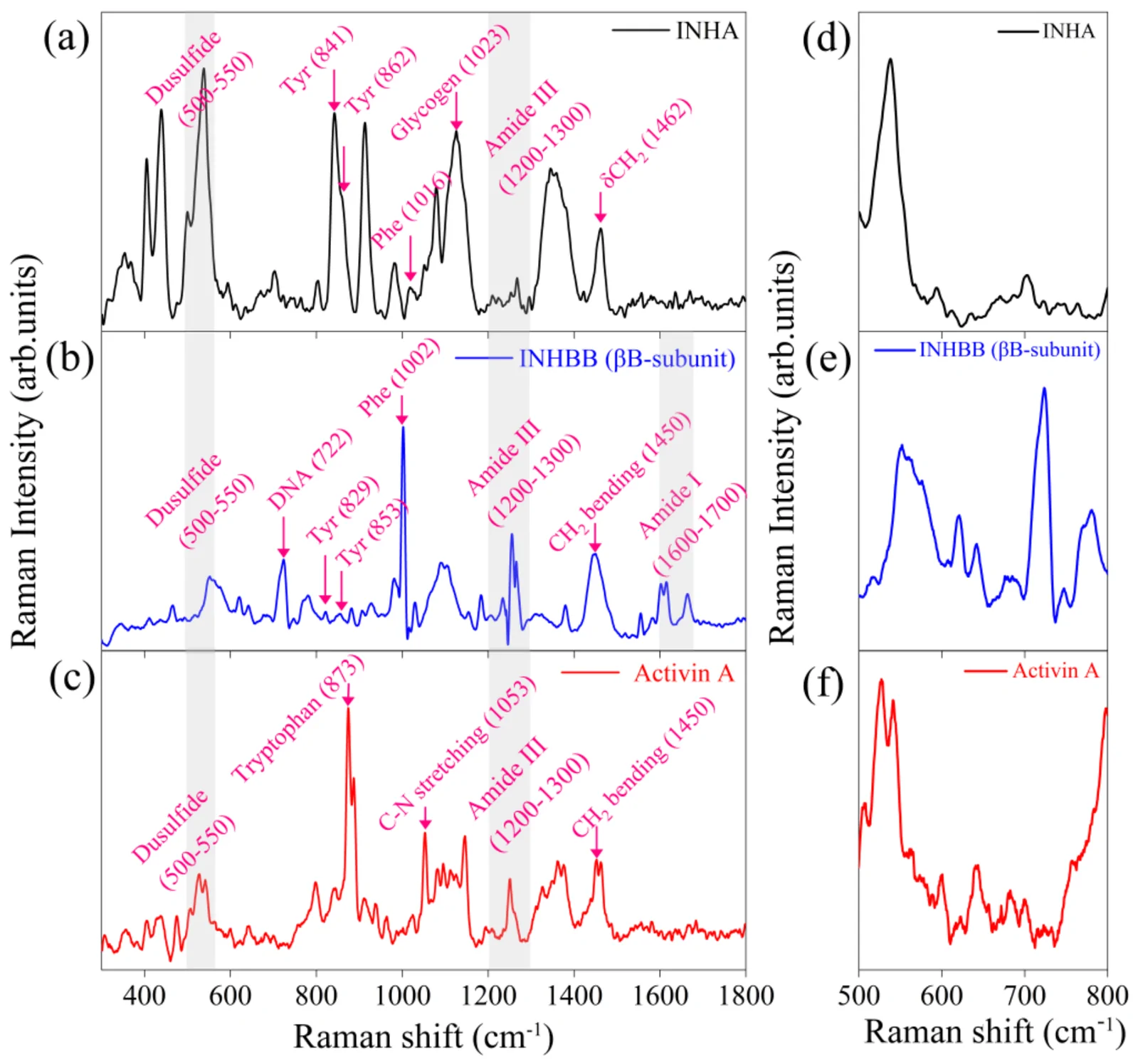
Comparative Raman spectra of recombinant human (**a**) inhibin α-subunit (INHA), (**b**) inhibin βB-subunit (INHBB (βB-subunit)) and (**c**) activin A (βA–βA homodimer). Spectra were baseline-corrected, Savitzky–Golay-smoothed, normalized, and vertically offset for clarity. (**d**–**f**) Zoomed-in Raman spectra of INHA, INHBB (βB-subunit) and activin A over the 500–800 cm^−1^ region to facilitate comparison of characteristic spectral features. Major Raman bands corresponding to the disulfide region (500–550 cm^−1^), tyrosine doublet (829/841/853/862 cm^−1^), phenylalanine (1002/1016 cm^−1^), amide III region (1220–1300 cm^−1^), and amide I region (1600–1700 cm^−1^) are labelled to facilitate interpretation of the molecular fingerprint [13,15,16,17,18,19,20,22,23,24,25,26,27,28,29,30,31,32,33,34].

**Table 1:** Assignments of characteristic Raman bands observed in recombinant TGF-β superfamily proteins. The peak assignments are based on established Raman spectroscopic studies of proteins and biomolecules.

Raman Shift (cm^−1^)	Molecular Vibration	Vibrational Mode	Structural Relevance in Proteins	References
500–550	S–S stretching vibrations	Disulfide bond stretching	Sensitive to the local disulfide bond environment and cysteine-containing structural motifs	[15,18,23,28,29,30,34]
830/850	Tyrosine residue vibrations	Ring-breathing-coupled and overtone vibration	Provides information on the local environment, hydrogen bonding, and conformational constraints of tyrosine residues	[15,18,24,26,27,31,32]
1003–1030	Phenylalanine ring-breathing vibrations	Aromatic ring breathing	Provides information on aromatic residue environment and serves as a characteristic protein marker band	[13,15,18,31,33]
1220–1300	Amide III vibrations	C–N stretching and N–H bending	Related to peptide backbone conformation and secondary structure organization	[15,18,29]
1600–1700	Amide I/C=C vibrations	Predominantly C=O stretching of peptide backbone. Aromatic C=C stretching may contribute	Provides information related to protein backbone conformation and secondary-structure composition	[15,18,29]

### 3.2. Comparison of Characteristic Raman Bands

To facilitate comparison, Raman bands related to protein structure were examined across the three proteins (Figure 1 and Table 1). Differences were observed in the disulfide stretching region (500–550 cm^−1^), where INHA exhibited the highest relative intensity. Variations were also evident in the aromatic amino acid residue regions, including the tyrosine doublet (~830–850 cm^−1^) and phenylalanine ring-breathing band (~1002–1016 cm^−1^), which may be associated with differences in the local environments of aromatic residues. Additional spectral variations were observed in the amide III (1220–1300 cm^−1^) and amide I (1600–1700 cm^−1^) regions, which primarily arise from peptide backbone vibrations. Although these characteristic regions were present to varying degrees in all three proteins, their relative intensities and spectral profiles differed, contributing to the overall molecular fingerprints. The normalized relative intensities of selected characteristic bands are summarized as a heatmap in Figure 2.

The numerical values within each cell represent the normalized relative intensity of the corresponding Raman band for each protein, with values closer to 1 indicating relatively higher intensity and values closer to 0 indicating relatively lower intensity after normalization. The accompanying color scale reflects the same relationship, with blue representing lower relative intensity and red representing higher relative intensity.

## 4. Discussion

The present study shows that recombinant human INHA, INHBB (βB-subunit), and activin A exhibit distinct Raman spectral signatures under the investigated experimental conditions, enabling their label-free differentiation based on their molecular vibrational features. Despite their related TGF-β superfamily architecture, the three proteins exhibited differences in Raman features, particularly in the disulfide-stretching, aromatic amino acid residue, and amide regions (Table 1). These differences may be associated with variations in protein composition and molecular organization. Measurements at multiple positions within each deposited sample further allowed us to assess the consistency of the Raman spectra across the sample.

The most pronounced spectral differences were observed in the disulfide-stretching region (500–550 cm^−1^), which is consistent with the cysteine-rich architecture of TGF-β family proteins (Figure 1 and Table 1 and Table 2). Members of this superfamily are stabilized by a conserved cystine-knot motif in which disulfide bonds contribute to the three-dimensional fold and, indirectly, to receptor recognition and biological activity [17,18]. Raman spectroscopy is also sensitive to S–S stretching vibrations, whose frequencies and intensities depend on disulfide conformation, dihedral angles, and the surrounding molecular environment, in addition to the number of cysteine residues [18,23,24,25,26]. Table 2 summarizes the Raman features identified across the disulfide, aromatic amino acid residue, amide III, and higher-wavenumber regions, which together contribute to the distinct spectral fingerprints of INHA, INHBB (βB-subunit), and activin A.

Accordingly, the differences observed among INHA, INHBB (βB-subunit), and activin A may be associated with differences in disulfide-bond environments arising from their distinct sequences and molecular organizations (Table 2). Because no independent structural characterization of disulfide geometry was performed, the observed spectral differences are considered consistent with differences in disulfide environments and do not provide direct evidence of specific disulfide conformations [18,24,26].

Variations were also observed in the amide I and aromatic amino acid residue regions. The amide I band arises predominantly from C=O stretching vibrations of the peptide backbone and has been widely used to provide information related to protein conformation and secondary-structure organization [15,21,24]. In the present study, the three proteins displayed different amide I profiles (Figure 1), which may be associated with differences in their local backbone environments and overall molecular organization. However, Raman spectra represent the collective vibrational response of the entire protein, and the present measurements do not provide direct quantitative estimates of individual secondary-structure elements. Similarly, differences in the tyrosine and phenylalanine regions may arise from changes in side-chain environment, hydrogen bonding, and molecular packing [15,22,24]. These assignments therefore may provide structural context for the observed fingerprints without directly measuring specific structural parameters.

The comparative heatmap (Figure 2) provides an overview of relative intensity differences in selected Raman regions related to protein structure. Differences in the S–S stretching, aromatic amino acid residue, and amide III regions contribute to the distinct spectral profiles of INHA, INHBB (βB-subunit), and activin A. These variations may represent molecular fingerprints under the experimental conditions used here, providing qualitative information about the proteins without directly quantifying individual structural parameters.

This proof-of-concept study has several important limitations. First, the proteins were examined in their commercially supplied forms, with INHA and activin A analyzed after lyophilization and INHBB (βB-subunit) analyzed in its storage buffer. These differences in sample state may influence Raman spectra and limit direct structural comparisons between the three proteins. Protein hydration affects hydrogen bonding, molecular flexibility, and conformational dynamics, while dehydration associated with lyophilization can alter protein–water interactions [15,30]. Accordingly, the reported spectra should be considered fingerprints of the specific sample states examined, providing information on the proteins under the experimental conditions used in this study. Second, the study included only three recombinant proteins and did not examine intact inhibin B (α–βB), activin B, activin AB, controlled protein mixtures, or complex biological matrices. Future work should incorporate replicate measurements from independently prepared samples, hydrated measurements, intact inhibin/activin dimers, additional TGF-β superfamily members, and controlled mixtures. For measurements under hydrated conditions or in low-concentration and complex biological samples, surface-enhanced Raman spectroscopy (SERS) may provide a complementary strategy for improving sensitivity, although optimization of surface chemistry will be necessary to minimize adsorption-induced conformational changes [27,28,34,35].

## 5. Conclusions

To the best of our knowledge, this study provides the first comparative Raman characterization of recombinant human INHA, INHBB (βB subunit), and activin A. The findings may suggest that Raman spectroscopy can capture molecular differences among closely related TGF-β superfamily proteins and may provide molecular and structure-related information complementary to conventional concentration-based assays. However, these Raman features do not directly measure protein conformation or individual secondary-structure elements. The present results should therefore be regarded as a proof of concept rather than evidence of diagnostic performance. Further studies using independently prepared samples, controlled hydrated conditions, intact inhibin/activin dimers, biologically relevant matrices, and adequately replicated datasets will be required to determine the broader applicability of Raman spectroscopy for molecular characterization and investigation of TGF-β superfamily proteins.

## Figures and Tables

**Figure 2:**
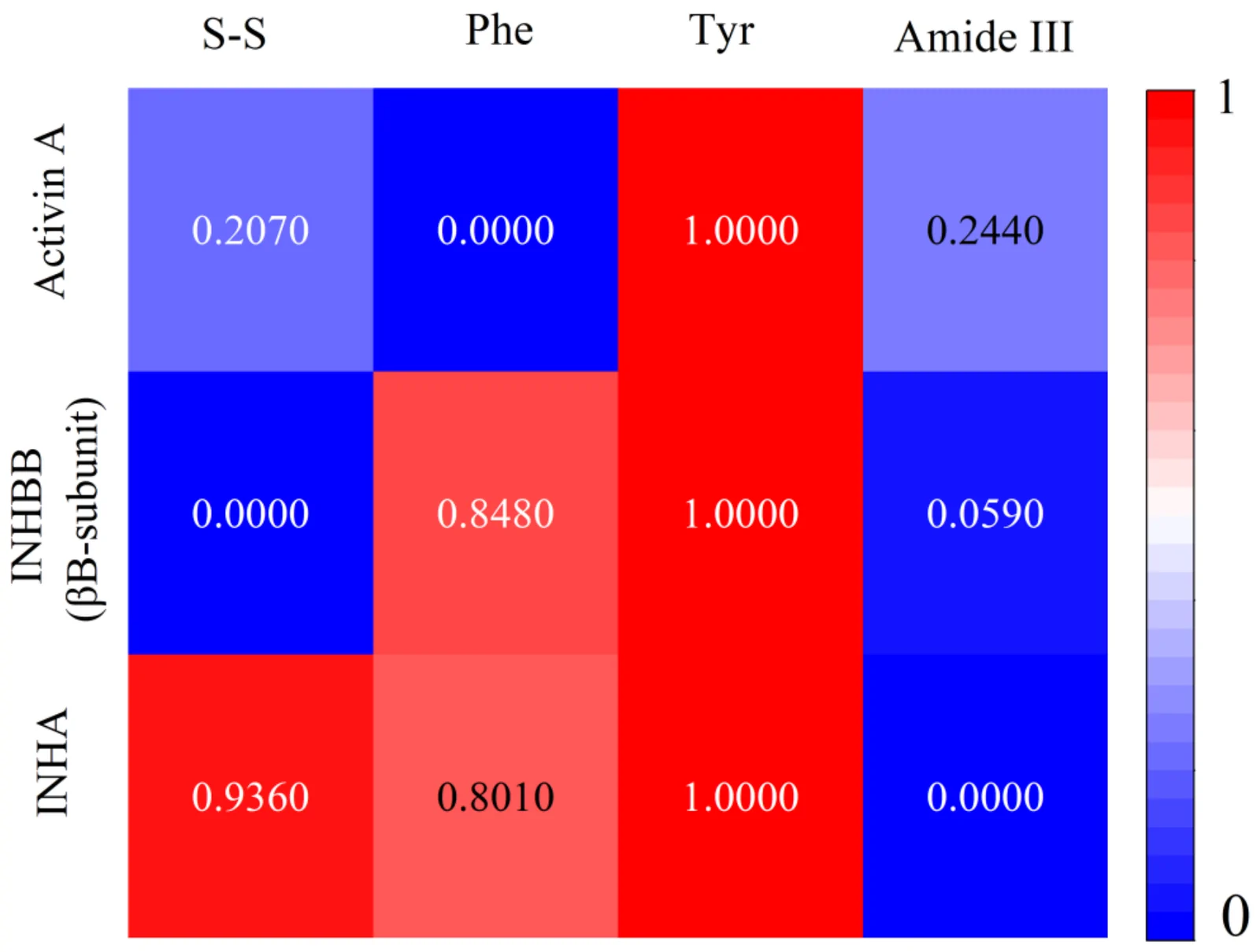
Heatmap showing the normalized relative intensities of representative Raman bands corresponding to S–S stretching (500–550 cm^−1^), tyrosine (~830–850 cm^−1^), phenylalanine (1002/1016 cm^−1^), and amide III (1220–1300 cm^−1^).

**Table 2:** Comparative Raman spectral features of recombinant human inhibin α-subunit (INHA), recombinant human inhibin B-βB-subunit (INHBB (βB-subunit)), and activin A (βA-βA homodimer).

Raman Shift (cm^−1^)	Peak Observed at			Possible Structural Origin	References
	INHA (cm^−1^)	INHBB (βB-Subunit) (cm^−1^)	Activin A (cm^−1^)		
500–550	498 and 538	548	528 and 538	Differences in disulfide bond environment related to distinct subunit composition and molecular organization	[18,24,26]
800–900	801 and 841/862	829 and 853	795 and 873	Variations in aromatic residue environment and local molecular interactions	[18,24,31]
1000–1030	1016 and 1023	1002	-	Differences in aromatic residue environment	[18,24,31]
1220–1300	1264	1254 and 1270	1247	Differences in peptide backbone organization	[18,24,26,28]
1350–1465	1341 and 1462	1382 and 1450	1365 and 1450	Differences in the local protein molecular environment	[18,24,26,27]
1600–1700	-	1608	-	Differences in backbone and aromatic vibrational contributions	[18,24,26]

## Data Availability

The original contributions presented in this study are included in the article. Further inquiries can be directed to the corresponding authors.
